# Implementing machine learning techniques for continuous emotion prediction from uniformly segmented voice recordings

**DOI:** 10.3389/fpsyg.2024.1300996

**Published:** 2024-03-20

**Authors:** Hannes Diemerling, Leonie Stresemann, Tina Braun, Timo von Oertzen

**Affiliations:** ^1^Center for Lifespan Psychology, Max Planck Institute for Human Development, Berlin, Germany; ^2^Thomas Bayes Institute, Berlin, Germany; ^3^Department of Psychology, Humboldt-Universität zu Berlin, Berlin, Germany; ^4^Department of Psychology, University of the Bundeswehr München, Neubiberg, Germany; ^5^Department of Psychology, Charlotte-Fresenius University, Wiesbaden, Germany

**Keywords:** machine learning (ML), emotion classification, audio emotion recognition, neural networks, speech signal features, Bilingual emotional classification

## Abstract

**Introduction:**

Emotional recognition from audio recordings is a rapidly advancing field, with significant implications for artificial intelligence and human-computer interaction. This study introduces a novel method for detecting emotions from short, 1.5 s audio samples, aiming to improve accuracy and efficiency in emotion recognition technologies.

**Methods:**

We utilized 1,510 unique audio samples from two databases in German and English to train our models. We extracted various features for emotion prediction, employing Deep Neural Networks (DNN) for general feature analysis, Convolutional Neural Networks (CNN) for spectrogram analysis, and a hybrid model combining both approaches (C-DNN). The study addressed challenges associated with dataset heterogeneity, language differences, and the complexities of audio sample trimming.

**Results:**

Our models demonstrated accuracy significantly surpassing random guessing, aligning closely with human evaluative benchmarks. This indicates the effectiveness of our approach in recognizing emotional states from brief audio clips.

**Discussion:**

Despite the challenges of integrating diverse datasets and managing short audio samples, our findings suggest considerable potential for this methodology in real-time emotion detection from continuous speech. This could contribute to improving the emotional intelligence of AI and its applications in various areas.

## Introduction

Non-verbal communication, including the different aspects of a speaker's voice, plays a crucial role in conveying emotions and is highly valued in interpersonal interactions. While verbal content is important, research suggests that humans are significantly influenced by non-verbal cues, even in purely acoustic expressions of emotion (Miller, 1981). In an increasingly globalized world, where technical means of signal transmission have become essential, understanding emotions through non-verbal cues gains even more significance (Morton and Trehub, 2001).

Research suggests that one intriguing question arising in this context is whether technical tools are capable of accurately predicting mood or emotions based on vocal parameters and acoustic measurements, independent of semantic content. If so, then this could allow for the analysis of convergences and divergences between verbal and non-verbal expressions, enriching communication in various contexts.

Previous scientific research used semantically closed audio recordings of roughly 1.5–5 s to develop classification tools (Chen et al., 2018; Jiang et al., 2019; Mustaqeem and Kwon, 2019, 2021; Mustaqeem et al., 2020). However, to apply such tools to dynamically measure change in emotions, algorithms to analyze audio recordings that are not semantically restricted are needed. The objective of this article is to develop such a classification tool that can recognize emotions in the voice. The tool is designed to process audio recordings in 1.5 s segments, identifying emotions regardless of the semantic content of the audio.

The decision to process audio recordings in 1.5 s segments merits further explanation. Implementing fixed time windows serves a dual purpose. Firstly, it simulates real-life scenarios where audio clips may be randomly segmented without any predefined understanding of when an emotion begins or ends. By establishing an algorithm that classifies emotions from these fixed segments, we are ensuring that the tool is robust enough to process audio in various real-world applications. Secondly, the use of shorter, fixed windows is strategically designed to minimize the likelihood of capturing multiple or mixed emotions within a single segment. This will attempt to ensure that the emotional content of each clip is as pure as possible when using real data in the future, which should lead to a more accurate classification.

Our rationale for selecting a 1.5 s window specifically has both empirical and practical origins. Empirically, the work of Lima et al. (2013) provided insights into the feasibility of emotion recognition from short non-verbal vocalizations. In their study, participants exhibited high accuracy in predicting emotions from audio clips that averaged around a second in length, suggesting that meaningful emotional content can be discerned from relatively brief snippets of sound. Practically, the choice of a 1.5 s window is consistent with the nature of our dataset. The dataset, composed of audios ranging from 1.5 to 5 s, contains emotionally charged but semantically neutral sentences. By opting for a 1.5 s segmentation, we can ensure that nearly every audio segment retains its original length without the need to artificially lengthen it with added silence. This approach essentially aims to extract the most emotionally salient part of each recording, which in part corresponds to the short vocalizations described by Lima et al. (2013).

This article will evaluate different machine learning techniques for the development of a robust tool capable of classifying emotions using these 1.5 s long audio clips. The effectiveness of this tool will be compared with the human ability to recognize emotions through voice. If the accuracy of the developed classifier is comparable to human judgment, it could not only serve practical applications but also allow researchers to infer aspects of human emotion recognition through reverse engineering.

### Decoding emotions

Contemporary emotion theories acknowledge the multidimensional nature of emotions, emphasizing their social and contextual aspects (Scherer, 2005; Fontaine et al., 2007; Moors et al., 2013). The tool presented in this article is based on Ekmans theory of basic emotions (Ekman, 1999). While Ekmans theory offers a practical and widely recognized framework, it is sometimes also criticized for its simplicity in representing human emotions. However, it provides a useful foundation for classifying emotions, while still allowing for a more nuanced understanding of emotions in future research.

Emotions, as dynamic processes, encompass several interrelated components. The diverse manifestations of emotions at various levels can be classified based on their distinct patterns of expression. This article uses the definition by Goschke and Dreisbach (2020) which includes all relevant parameters, giving a holistic picture of the multifaceted nature of emotions:

“Emotions are psychophysical reaction patterns based on more or less complex evaluations of a stimulus situation, which are accompanied by a series of peripheral physiological changes as well as the activation of certain central nervous systems. These reactions motivate certain classes of behavior, can be expressed in specific facial expressions and body postures, and are often (but not necessarily) associated with a subjective quality of experience” (Goschke and Dreisbach, 2020).

This article follows the assumption that emotions, despite their nature as dynamic processes consisting of multiple components, can be assigned to categorize based on their patterns of expression. This assumption follows the concept of basic emotions, which Scherer (1985) recognizes as the main types of emotions. Ekman (1999) specifies the seven basic emotions as fear, surprise, anger, disgust, joy, sadness, and contempt, which have universal characteristics and are intuitively performed and also recognized by humans.

The ability to recognize and classify emotions is called cognitive empathy. Not every emotion is recognized equally well, as cognitive empathy is a combination of many subskills with interpersonal and intrapersonal differences (Marsh et al., 2007). In a conversation, not only linguistic cues are used to recognize emotions, but also non-verbal paralinguistic cues. Paralinguistic signals accompany what is spoken, for example, speaking rate or volume, and expands the spoken words with additional aspects that provide information about the speaker's state of mind (Bussmann and Gerstner-Link, 2002).

### Emotions in the voice

The facial and vocal expression of basic emotions are understood cross-culturally, and these emotions are associated with similar physiological patterns of change (Ekman et al., 1983). These emotions are also universally recognized through vocal expression (Izdebski, 2008). The human voice serves as a powerful channel for expressing emotional states, as it provides universally understandable cues about the sender's situation and can transmit them over long distances. Voice expression is rooted in brain regions that evolved early in human development, underscoring its fundamental role in our evolutionary history (Davitz, 1964; Morton, 1977; Jürgens, 1979).

When categorizing emotions based on vocal expressions, employing a limited number of emotion categories proves advantageous to avoid overwhelming information (Johnson-Laird and Oatley, 1998). Additionally, distinct emotion specific patterns of acoustic features have been observed (Scherer, 1979), which can still be detected even after removing linguistic cues from the speech signals. Physiological parameters significantly influence vocal parameters like loudness, fundamental frequency, noise components, and timbre (Trojan et al., 1975; Frick, 1985; Burkhardt, 2000).

### Related publications

Several classification tools have been developed to recognize and classify emotions in the voice. A notable example is Xiaos classifier, which utilizes artificial neural networks and incorporates pre-classification to enhance accuracy (Xiao et al., 2010). More recent developments have focused on convolutional neural networks (CNNs) and their ability to efficiently process large amounts of data (Chen et al., 2018; Jiang et al., 2019; Mustaqeem and Kwon, 2019, 2021; Mustaqeem et al., 2020). For instance, the study by Mustaqeem and Kwon (2021) introduces a complex two-stream CNN that achieves high accuracies for different emotion databases. Table 1 presents the performance metrics of various classification tools as reported in the cited studies, which utilize differing methodologies:

**Table 1:** Classifier performance of studies using Emo-DB and RAVDESS databases.

**Referenes**	**Method^a^**	**DB^b^**	**Perf**.
Xiao et al. (2010)	NN-PC	E	81.2%
Chen et al. (2018)	CNN	E	82.8%
Jiang et al. (2019)	CNN	E	84.5%
Mustaqeem et al. (2020)	CNN	E	85.5%
Mustaqeem et al. (2020)	CNN	R	77%
Mustaqeem and Kwon (2019)	CNN	R	79%
Mustaqeem and Kwon (2021)	2S-CNN	E	95%
Mustaqeem and Kwon (2021)	2S-CNN	R	85%

This table provides an overview of accuracies achieved by various studies.

a Method: NN, Neural Network with pre-classification (PC); CNN, Convolutional Neural Network; 2S-CNN, two-Stream Convolutional Neural Network.

b DB: E, Emo-DB; R, RAVDESS.

1. Xiao et al. (2010) employ a 10-fold cross-validation method with a 50:50 train-test split for each fold and include a preclassification step to determine gender. The table lists their reported average accuracy.

2. Chen et al. (2018) implement a 10-fold cross-validation, splitting the data for each split by speakers: eight for training, one for testing, and one for validation, targeting four emotional states happy, angry, sad, and neutral. The corresponding average accuracy figures are depicted in the table.

3. Jiang et al. (2019) adopt a Leave-One-Speaker-Out (LOSO) approach. Shown in the table is the unweighted average accuracy accumulated across all trials.

4. Mustaqeem et al. (2020) use a 5-fold cross-validation, designating eight speakers for training and two for testing in each fold. The table illustrates their average accuracy results.

5. Mustaqeem and Kwon (2019) execute a 5-fold cross-validation with an 80:20 split for training and testing, respectively. Their average accuracy is shown in the table.

6. Mustaqeem and Kwon (2021) perform a 10-fold cross-validation with an 80:20 train-test split, with the table showing the F1 scores as the most relevant performance parameter, as presented in the referenced source.

However, it is important to note that the performances outlined above cannot be directly compared with the results of this article. Firstly, the methodologies employed across these studies vary. Secondly, the databases used are also distinct, given that this study utilizes audio clips trimmed to 1.5 s as opposed to complete audio recordings. In particular, we aim to demonstrate that emotion recognition based on voices, when using the right tools, is also possible when using very short time segments, which can be used for continuous emotion classification of voice data. The performances shown are intended to provide an overview of the existing classifiers that have been trained on the data used here in order to be able to better contextualize this article.

All the aforementioned approaches utilize audio recordings from the Emo-DB and the RAVDESS databases. These databases offer clearly recognizable emotion recordings in complete sentences or uniformly defined speech units, which has led to limited attention being given to audio segmentation in previous research. However, the challenge lies in spontaneous speech where defining unambiguous units becomes difficult. An effective segmentation approach needs segments long enough to extract acoustic patterns but also short enough to capture emotional state changes. Studies on continuous segmentation have already been undertaken in the literature. Atmaja and Akagi (2020) showed emotion recognition beyond chance for a visual-auditory dataset using a 4 s time window.

Contrary to the studies mentioned above, the work of Stresemann (2021) takes a different approach. She standardized all audio recordings from these databases to a length of 1.5 s, analyzing them as independent units without considering the grammatical sentence structure. The aim is to focus purely on emotion recognition, disconnecting it from the semantic content of the sentences. This choice of approach, which sometimes results in the cropping of longer files and the potential loss of words, is supported by Scherer (1979). He argued for the existence of emotion-specific acoustic patterns that are independent of contiguous sequences. This approach not only aids in mapping emotion expression changes within longer sentences but also has a practical benefit: it is especially applicable in online settings where smaller datasets can be quickly analyzed, and reliable assessments can be made.

### Approach of this study

This article aims to enable automatic continuous classification by limiting the duration of individual audio segments to 1.5 s. The practical objective is to continuously split a longer audio track into potentially overlapping sequences, allowing the model to provide a continuous assessment of emotions in the voice. The study by Stresemann (2021) serves as the foundation for this article, but the approach here uses a more automated method with advanced machine learning techniques. The fixed time length of 1.5 s is intended to simulate the challenges in real-life datasets. Using audio files of different lengths would require upstream recognition in real data. The specific length of 1.5 s serves as a compromise between the shortest possible audio length to avoid overlapping emotions and enough information to still allow humans to understand the audio files. The aim of this study is to proof that automatic classification of human speech is possible under these constraints. Thereby, we aim to show that a tool can be created, which automatically classifies emotions in continuous human speech, without the necessity of elaborate preprocessing. To do so, we present an approach that compares different model designs and different combinations of linguistically diverse audio tracks in terms of their accuracy in emotion recognition both to each other and to humans.

## Methodology

Building on the theoretical background outlined earlier, this section delves into the methodology employed in this study. The processing of the audio data is discussed first, followed by a detailed explanation of the datasets and a comparison with human performance. The latter part of this section will describe the generation of individual features and the development and testing of various models.

### Audio

The audio material for this study was sourced from two publicly accessible emotion databases from distinct cultures: Germany and Canada. This choice is grounded in the cross-cultural universality of emotions in audio, as supported by the meta-analysis conducted by Juslin and Laukka (2003). The considered emotions for this study include joy, anger, sadness, fear, disgust, and neutral.

Specifically, English-language recordings were extracted from the Ryerson Audio-Visual Database of Emotional Speech and Song (RAVDESS; Livingstone and Russo, 2018). An example of content from RAVDESS is the neutral statement, “Dogs are sitting by the door.” For German-language recordings, the Berlin Database of Emotional Speech (Emo-DB) was used (Burkhardt et al., 2005). A representative sentence from Emo-DB is “Der Lappen liegt auf dem Eisschrank” (the rag lies on the refrigerator). In both databases, actors induced the emotions using emotional memory techniques.

For the audio processing stage, we settled on a strategic duration of 1.5 s per segment. This choice was influenced by several factors: to emulate real-world conditions where snippets of emotion may lack clear starting or ending points, to approximate the briefest discernible emotional span, and to minimize the potential for overlapping emotions in a single clip. Files longer than this were trimmed to capture the core 1.5 s, with any excess equitably truncated from both the start and end. Conversely, shorter files were symmetrically extended with silence on both sides, ensuring a consistent segment length while preserving the original emotional content. In other studies (e.g., Chen et al., 2018; Jiang et al., 2019; Mustaqeem and Kwon, 2019; Mustaqeem et al., 2020), the audio files were not segmented. In order to additionally examine whether and how much accuracy is lost due to the selected length of the segmentation of the audio recordings, audio files that were segmented to 3 or 5 s were also used for parts of the utilized model designs. The same segmentation method was used for all variants.

#### The Ryerson Audio-Visual Database of Emotional Speech and Song

The RAVDESS is an open-access database offering 7,256 English-language recordings, both spoken and sung, spanning across three modalities: audiovisual, video-only, and audio-only (Livingstone and Russo, 2018). For the purpose of this study, only the audio modality was employed. Featuring recordings from 24 actors (12 male, 12 female), the database represents six emotions (joy, sadness, anger, fear, surprise, and disgust) in addition to two baseline states (neutral and calm). From RAVDESS, this research incorporated 1,056 audio clips, omitting the emotions of surprise and calm, each trimmed to a precise duration of 1.5 s.

#### Berlin Database of Emotional Speech

The Emo-DB, hosted by the Technical University of Berlin, is a public database comprising 535 German-language recordings, conducted by 10 actors (five male and five female) under the guidance of phoneticians (Burkhardt et al., 2005). The database encompasses the emotions of anger, fear, joy, sadness, disgust, and neutral speech. From the Emo-DB, 454 recordings were incorporated into this study, with the emotion of surprise excluded, and every clip was trimmed to 1.5 s.

#### Comparison to human performance

The data format for this research aligns with the methodology of Stresemann (2021), involving 61 participants (36 male and 25 female) aged between 20 and 71 years. Participants were tasked with a forced-choice format survey where they matched emotions to 82 English language recordings from the RAVDESS database and 88 German recordings from the Emo-DB. Covered emotions were fear, anger, joy, sadness, disgust, and neutral speech.

Before starting, participants received comprehensive information regarding the study procedure, data privacy guidelines, and the voluntary nature of participation. The survey also collected demographic details, including sex, age, first language, current domicile, and prior experience in English-speaking regions. The listening exercise required a quiet environment, where participants identified emotions immediately after a single playback. In cases of unclear recordings due to technical issues, an alternative “no statement” option was available. All data, barring one problematic disgust recording, were included in the final analysis.

The findings of Stresemann (2021) revealed a robust positive correlation between recognition rates on the Emo-DB and RAVDESS databases, indicating that individual empathic abilities might supersede linguistic or cultural biases in emotion recognition. This correlation is possibly influenced by the shared Germanic roots of English and German, leading to similarities in fluency and intonation. Conversely, studies contrasting different linguistic backgrounds highlighted advantages for listeners when the recordings matched their native tongue. For instance, native English speakers surpassed Spanish and Japanese counterparts in emotion recognition (Graham et al., 2001). Similarly, Korean speakers outdid their French and American peers when classifying emotions in Korean (Chung, 2000).

While basic emotions' expression is broadly universal, nuances exist due to cultural differences (Graham et al., 2001). However, numerous studies, such as Juslin and Laukka (2003), underscore high cross-cultural emotion recognition rates. This suggests that even amidst cultural distinctions in emotional expression, humans' inherent auditory-driven emotion recognition abilities transcend linguistic and cultural confines. This inherent capability, albeit less refined than facial emotion recognition, does not necessitate formal training or guidance.

#### Feature generation

Once audio recordings were streamlined into 1.5 s segments, we embarked on generating a diverse set of features. The ambition was to mine maximum information through various methodologies, ensuring redundancy was at its minimal.

The following is an overview of the individual features created in this study. Each feature was calculated for each audio recording. For some features, summary values for the 1.5 s were computed (e.g., the mean for pitch). Table 2 gives an overview of all features together with the number of data points this feature provides. The “Summarization” column describes the summary approach for each variable, if one was used. Overall, there were 14,244 different entries for each audio recording. Given the potentially multidimensional nature of expressing each emotion in the voice, preselecting features could result in information loss. Therefore, the approach in this study was to generate as many features as possible, allowing the models to independently select relevant features. The features used here include:

**Table 2:** Enumeration of dataset features, summarization, and quantity.

**Feature**	**Summarization**	**Quantity**
Unmodified Audio Signal	Variance	1,200
HPSS	Variance	2,400
Spectral Flatness	N/A	47
Spectral Centroid	N/A	47
Fundamental Frequency	N/A	47
Spectral Rolloff	N/A	94
Spectral Bandwidth	N/A	47
Zero Crossing Rate	N/A	47
Root Mean Square	N/A	47
Spectral Contrast	N/A	188
Tonnetz	N/A	282
Chroma	N/A	564
Pitch Tracking	Var. and mean^*^	2,050
Pitch Magnitudes	Var. and mean^*^	2,050
Magnitude	Var. and mean^*^	2,050
Phase	Var. and mean^*^	2,050
MFCC	N/A	940

Features, summarization, and quantity for the dataset.

* Variance and mean calculated for each 2,048 Hz window.

##### Unmodified Audio Signal

The Unmodified Audio Signals served as the foundation for all subsequent feature calculations. A portion of the signal was preserved to retain potential unbiased information that may not be captured by other features.

##### Spectral flatness

Spectral Flatness is a measure of how evenly the energy of an audio signal is distributed across different frequency bands compared to a reference signal. It provides an estimate of the flatness of the signal and may be associated with certain emotions (Dubnov, 2004).

##### Spectral centroid

The Spectral Centroid indicates the average frequency at which the energy of a sound signal is centered. It can be used to estimate the perceptual brightness or tonal brightness of the sound and is sometimes related to valence and arousal, which are closely connected to emotions (Klapuri and Davy, 2007).

##### Fundamental frequency

Fundamental Frequency (F0) estimation means determining the lowest frequency and rate of periodicity in a sound signal. Analyzing the F0 provides information about the emotional dimensions of the signal (Cheveigna and Kawahara, 2002; Mauch and Dixon, 2014).

##### Voiced

In addition to F0 estimation, the presence of a voice within a specified time window of the audio was measured, along with the probability of voice presence. The specific time window used was 2,048 Hz (Cheveigna and Kawahara, 2002; Mauch and Dixon, 2014).

##### Spectral rolloff

Spectral Rolloff indicates the frequency level at which a certain percentage (here, 0.85) of the energy is contained in the signal. It can identify the frequency ranges that are most strongly represented in the signal and may aid in emotion recognition (Sandhya et al., 2020).

##### Pitch tracking

Pitch Tracking estimates the pitch or fundamental frequency (F0) of a sound signal and measures its magnitude. This feature can provide additional information related to the F0 and assist in emotion classification (Smith, 2011).

##### Harmonic percussive source separation

The HPSS technique separates a sound signal into its harmonic and percussive components. Both components could convey different emotional information (Fitzgerald, 2010; Driedger and Müller, 2014).

##### Magphase

Magphase separates the complex-valued spectrogram (D) into its magnitude (S) and phase (P) components, where D = S × P. The magnitude is used to calculate various emotion-related features presented in this section, while the phase angle is measured in radians and used as is. The phase encodes relationships between different frequency components of the signal, which may contain emotional information, although it is rarely used in emotion classification (Librosa Development Team, 2023).

##### Spectral bandwith

Spectral Bandwidth is a measure of the spread of the spectral content of the signal. It is related to the frequency range of the signal and may be relevant to emotions (Klapuri and Davy, 2007).

##### Spectral contrast

Spectral Contrast refers to the differences in energy levels between different frequency ranges of an audio signals. It can describe the tone color of a signal, which might be associated with certain emotions (Jiang et al., 2002).

##### Zero crossing rate

The Zero Crossing Rate indicates the number of times the signal changes from positive to negative or vice versa. It can provide information about the dynamics of the signal (Hung, 2009).

##### Mel-frequency cepstral coefficients

MFCC are widely used features in music and speech recognition. They represent the Mel-requency energy distribution of an audio signal and can identify the most important frequencies of the signal while being robust to changes in loudness and sound characteristics (Sato and Obuchi, 2007).

##### Root mean square

RMS is a measure of the average power of an audio signal. It indicates the average loudness of the signal and can describe its loudness level (Chourasia et al., 2021).

##### Tonnetz

Tonnetz is another representation of frequency ranges that can be used to identify the harmony of a musical signal, which might be associated with certain emotions (Harte et al., 2006).

##### Chroma

Chroma represents the presence of different frequency ranges in a music signal and can be used to identify the key of the music signal, potentially containing emotion-related information (Ellis, 2007).

##### Creation of the spectrograms

Spectrograms visually depict the frequency spectrum of audio signals, reflecting energy distribution across time and frequency. Such patterns have been identified as crucial in emotion recognition (Kim et al., 2010). For our study, spectrograms were crafted for every audio recording, saved as PNGs (without axes or borders) at a resolution of 320 × 240 pixels.

Subsequently, we detail the employed classification models.

#### The deep neural network

DNNs, renowned for their prowess in intricate pattern recognition, consist of interconnected feedforward layers with varying neuron counts (LeCun et al., 1998). The architecture allows the model to adjust to input data, predicting emotions via gradient-based learning.

#### The convolutional neural network

The generated spectrograms consist of numerous data points, resulting in 230,400 data points (320 × 240 × 3) for each image. To efficiently analyze these images, CNNs are employed. These networks, skilled at image processing through local receptive fields and weight sharing, enhance the representation using pooling, particularly max pooling, to retain essential data while reducing the image size (LeCun and Bengio, 1995).

#### The hybrid model C-DNN

Our hybrid C-DNN model merges the insights of both the generated features and spectrograms. It encompasses a dual-input approach: a DNN for feature processing and a CNN for spectrogram analysis. The output layers from both networks converge into a concatenated layer, followed by another feedforward DNN predicting emotions through a softmax function. The goal is to determine whether combining spectrograms and features improves information extraction compared to individual data sources.

#### Creation of the models

The three model designs described above were implemented in a Python environment using Tensorflow (Abadi et al., 2015) and Scikit-learn (Pedregosa et al., 2011). The dataset was apportioned into training (80%) and test sets. The hyperparameters for each model were defined separately using Bayesian optimization with a Gaussian process based on the associated training dataset. A brief overview of the hyperparameter is listed in Table A1. Using Bayesian optimization, different models were formed, their hyperparameters adjusted, and subsequently trained on the training dataset. Post every training epoch, the test dataset underwent a prediction process. After completing up to four training epochs, validation accuracy was gauged a final time. The validation accuracy from the test data was then used as a benchmark to avoid overfitting.

#### Testing the different models

For a more consistent comparison with existing literature, the models underwent a 10-fold cross-validation.

The performance metrics employed to measure the model quality included Balanced Accuracy (BAC). This was compared to both random classifications and the BAC achieved by other models.

Our evaluation approach combined Independent Validation (Kim and von Oertzen, 2018) with Bayesian Updating. Initially, models were trained on 10% of the total data, setting aside another 10% for validation, ensuring overfitting was kept within limits. The models were then sequentially introduced to new data in chunks of 16 data points. Before integrating these data points into the primary training dataset, the models attempted their prediction, updating the BAC's posterior distribution via Bayesian techniques. This cyclic procedure continued until the entire dataset had been incorporated into the training set, with the validation set consistently monitoring for overfitting.

Successful and unsuccessful predictions were used to update the parameters of a beta distribution through Bayesian Updating, providing a posterior distribution of the classifier accuracy. A beta distribution was chosen to model the accuracies as it can depict that a perfect accuracy of 1 is very unlikely or even impossible, while other values can be equally likely. By comparing the overlap between the beta distributions of the models, one could assess the probability of one model outperforming another, for instance, a classifier that merely guesses the results. This statistical approach allowed us to validate the effectiveness and generalizability of our model while providing a measure of uncertainty.

#### Testing against humans

To evaluate the performance of human participants, we used a similar approach, assuming a binomial distribution for the correct recognition of emotions. We then estimated the accuracy using a beta distribution. By comparing the overlaps among the distributions for each emotion, we can determine the similarity in performance and assess the likelihood of differences between human participants and the classifiers.

## Results

This section presents the outcomes from the model comparisons. First, we compared the models using cross-validation. For all following Bayesian accuracy estimations, we used a beta(1,1) prior, which stands as the conjugate prior for a binomial distribution, representing minimal prior information.

### Cross validation

**Figure 1:**
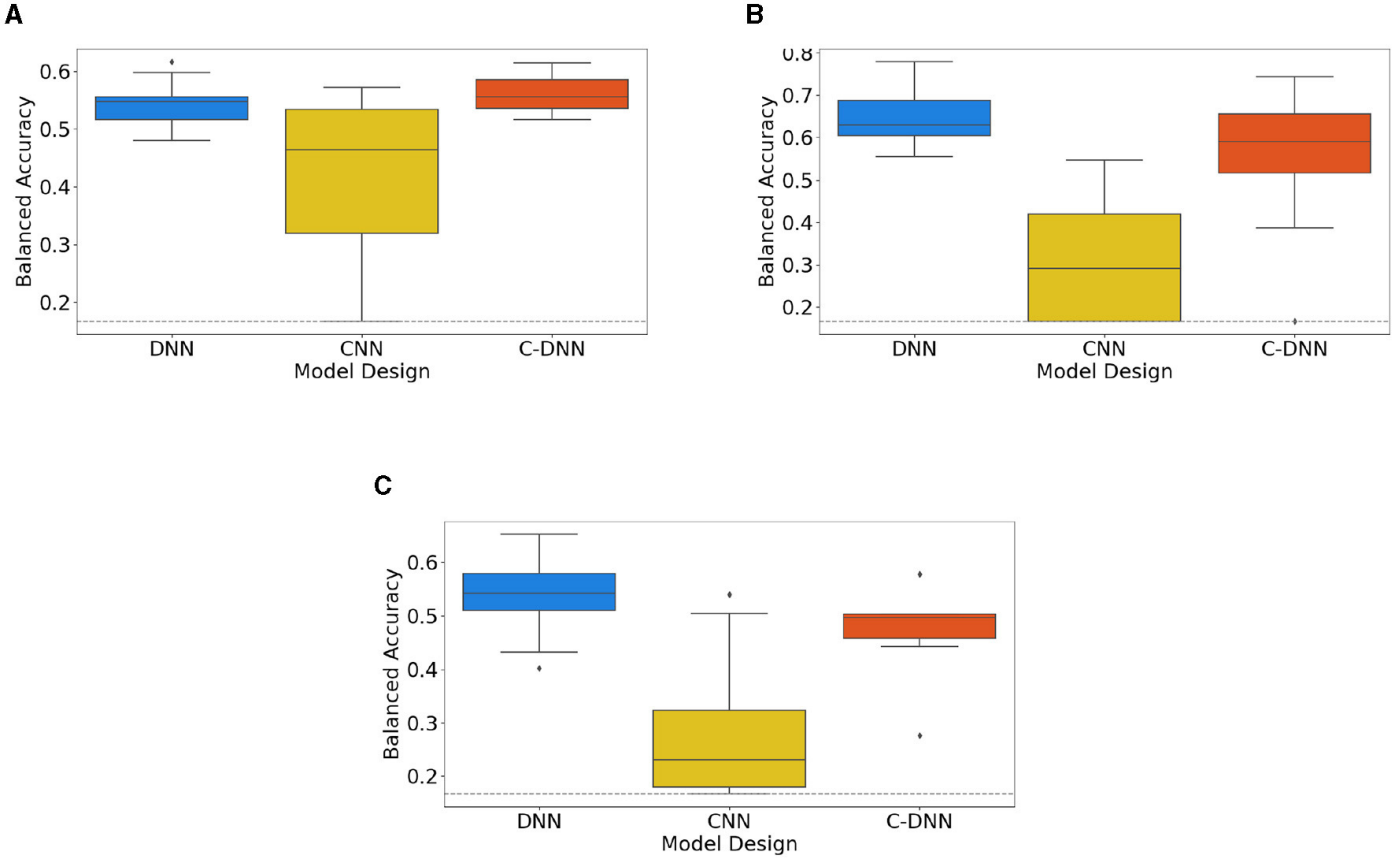
Neural network design (DNN, CNN, and C-DNN) comparisons based on cross-validation results. This figure shows the results of 10-fold cross-validations for the comparison between different neural network designs (DNN, CNN, and C-DNN) based on both combined and separate datasets. Subfigures represent: **(A)** results based on the combined Dataset, **(B)** results based on Emo-DB, and **(C)** results based on RAVDESS. The gray dashed line indicates the Balanced Accuracy of a random classifier.

Figure 1 presents the outcomes of 10-fold cross validations for three distinct model designs, individually trained on different datasets: the combined dataset in A, the Emo DB dataset in B, and the RAVDESS dataset in C, respectively. The boxplots illustrate the model performances, offering a visual comparison across the diverse model designs and datasets. The corresponding mean values for each model design, computed from the different datasets, are consolidated in Table 3, thereby facilitating a numerical evaluation of the model performances. For the model design DNN, additional models are created based on 3 and 5 s segmented audio files. This results for the combined dataset in 62.36% (3 s) and 61.79% (5 s). For the Emo-DB dataset, the results are 72.91% (3 s) and 69.21% (5 s). Results for the RAVDESS dataset are 60.01% (3 s) and 61.00% (5 s).

**Table 3:** The mean of balanced accuracies of various models based on 10-fold cross-validation.

**Dataset**	**DNN**	**CNN**	**C-DNN**
Combined	54.49%	41.56%	56.24%
Emo-DB	64.69%	30.68%	54.85
RAVDESS	53.55%	28.39%	48.09%

DNN, Deep Neural Network; CNN, Convolutional Neural Network; C-DNN, Combination of Deep Neural Network and Convolutional Neural Network.

### Combined dataset

**Figure 2:**
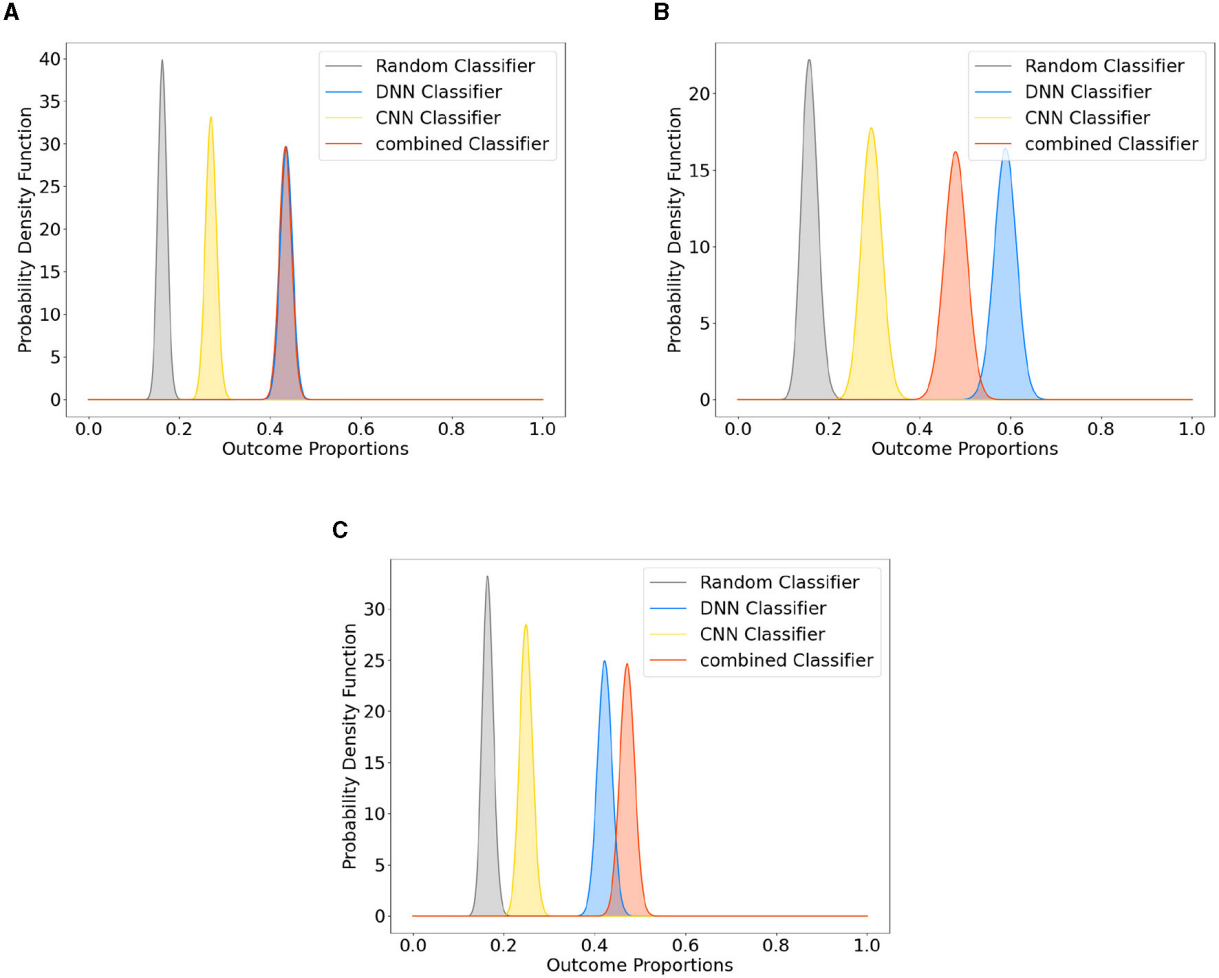
Posterior distributions of neural network designs (DNN, CNN, and C-DNN) vs. a random classifier. This figure demonstrates the posterior distributions for different neural network designs (DNN, CNN, and C-DNN) compared to a random classifier. Subfigures represent: **(A)** Classifier comparison based on the Combined Dataset, **(B)** Classifier comparison based on Emo DB, and **(C)** Classifier comparison based on RAVDESS.

**Figure 3:**
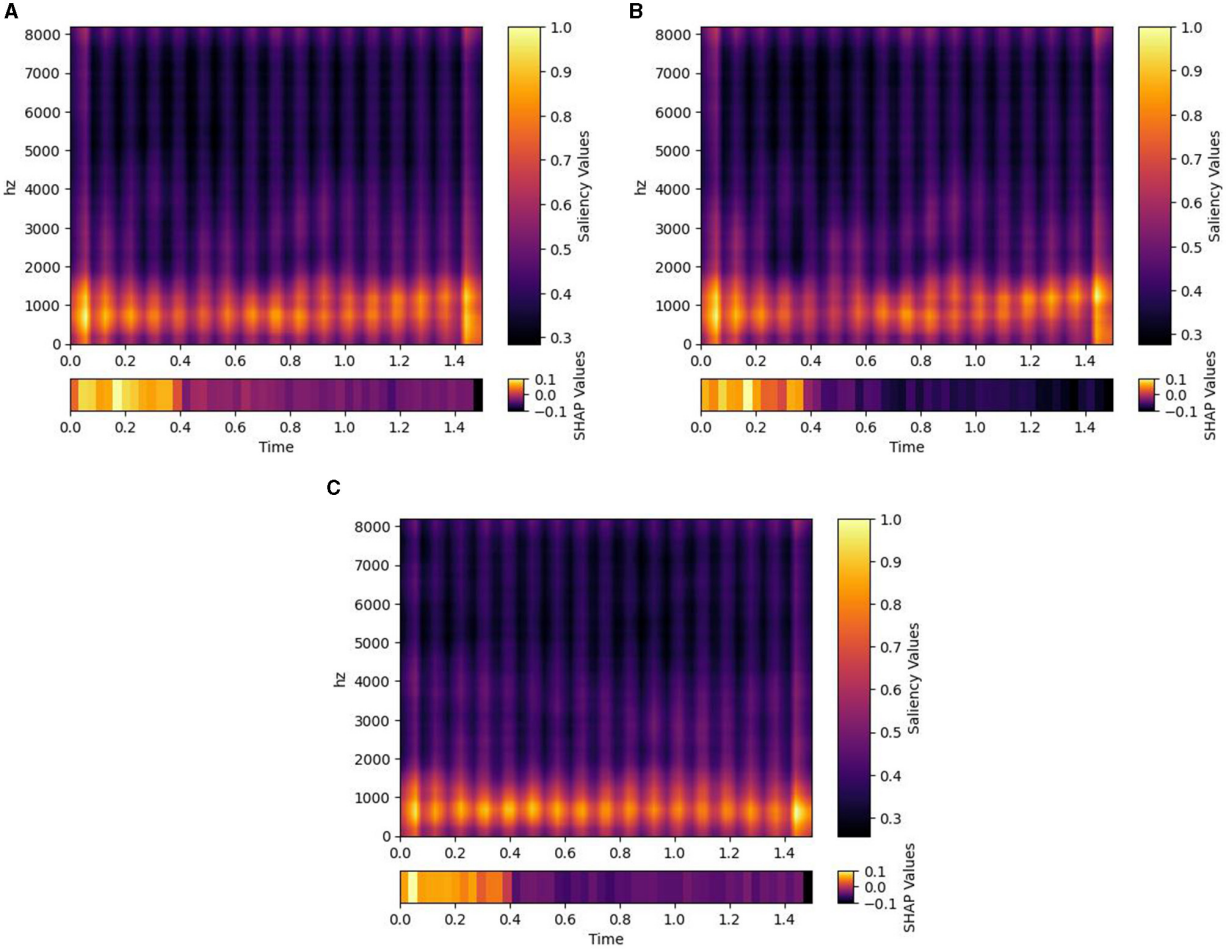
Saliency maps and SHAP values for different datasets. Each plot illustrates the average saliency across all spectrograms derived from emotional audio recordings within their respective datasets. **(A)** represents the combined dataset, **(B)** the Emo_DB dataset, and **(C)** the RAVDESS datasets. The color gradient within each plot signifies varying saliency values, while the bar beneath it provides SHAP values for 48 time segments, indicating the significance of individual features grouped by time intervals.

Figure 2 presents the results obtained from the Bayesian estimate of the classifier accuracies. The three different model designs, that is, DNN, CNN, and combined, all trained on the Emo DB and RAVDESS datasets combined. The posterior distribution of each classifier is shown alongside the posterior distribution of random classification. The posterior distributions indicate where the true performance under each classifier is expected to be. A distribution closer to the maximum value of 1 indicates a better performance. The posterior distribution of the random classifier (indicating guessing) is to the left of the posterior distributions of the trained classifiers and only overlapps by 1%. This indicates that the probabilbiity that the classifiers perform better than guessing is above 99%. The position of the distributions is described by the maximum a-posteriori estimate (MAP), the peak of the posterior distribution. The MAP performance of two of the models (DNN and C-DNN) is close to 0.45 (0.436 and 0.433) with a standard error of 0.013. The CNN model performance is lower compared to the other models (0.27) with a standard error of 0.012. Note that with six categories to classify, guessing performance is 1/6. Analysis of the average saliency maps across all spectrograms obtained from the Emo DB, RAVDESS, and combined datasets has provided insights into the time-segment relevance for emotion classification. As depicted in Figure 3, the distribution of SHAP values across 48 time segments reveals variations in the predictive importance of certain time intervals. Notably, segments with higher SHAP values indicate a stronger influence on model predictions, which suggests that certain temporal portions of the audio recordings are more salient for emotion detection.

### Emo DB separated

To further investigate the performance of the Emo DB and RAVDESS datasets separately, a corresponding method was used to compare them to a random classifier. The corresponding results for the Emo DB Dataset are shown in Figure 2B. The analysis shows that assuming a flat prior, the probability of the models on these datasets differing from a random classifier is over 99%. The posterior distribution of each classifier is shown alongside the posterior distribution of random classification. The MAP performance of two of the models (DNN and C-DNN) is close to 0.5 (0.58 and 0.48) with a standard error of 0.024. The CNN model performance is lower than the other models (0.29) with a standard error of 0.022.

### RAVDESS separated

The corresponding results for the RAVDESS Dataset are shown in Figure 2C. The posterior distribution of each classifier is shown alongside the posterior distribution of the random classification. The probability that the classifier performs better than guessing is above 99% throughout. The MAP performance of all three models (DNN, CNN and C-DNN) is close to 0.5 (0.42 and 0.42) with a standard error of 0.016. The CNN model performance is lower than the other models (0.26) with a standard error of 0.014.

### Comparison to humans

**Figure 4:**
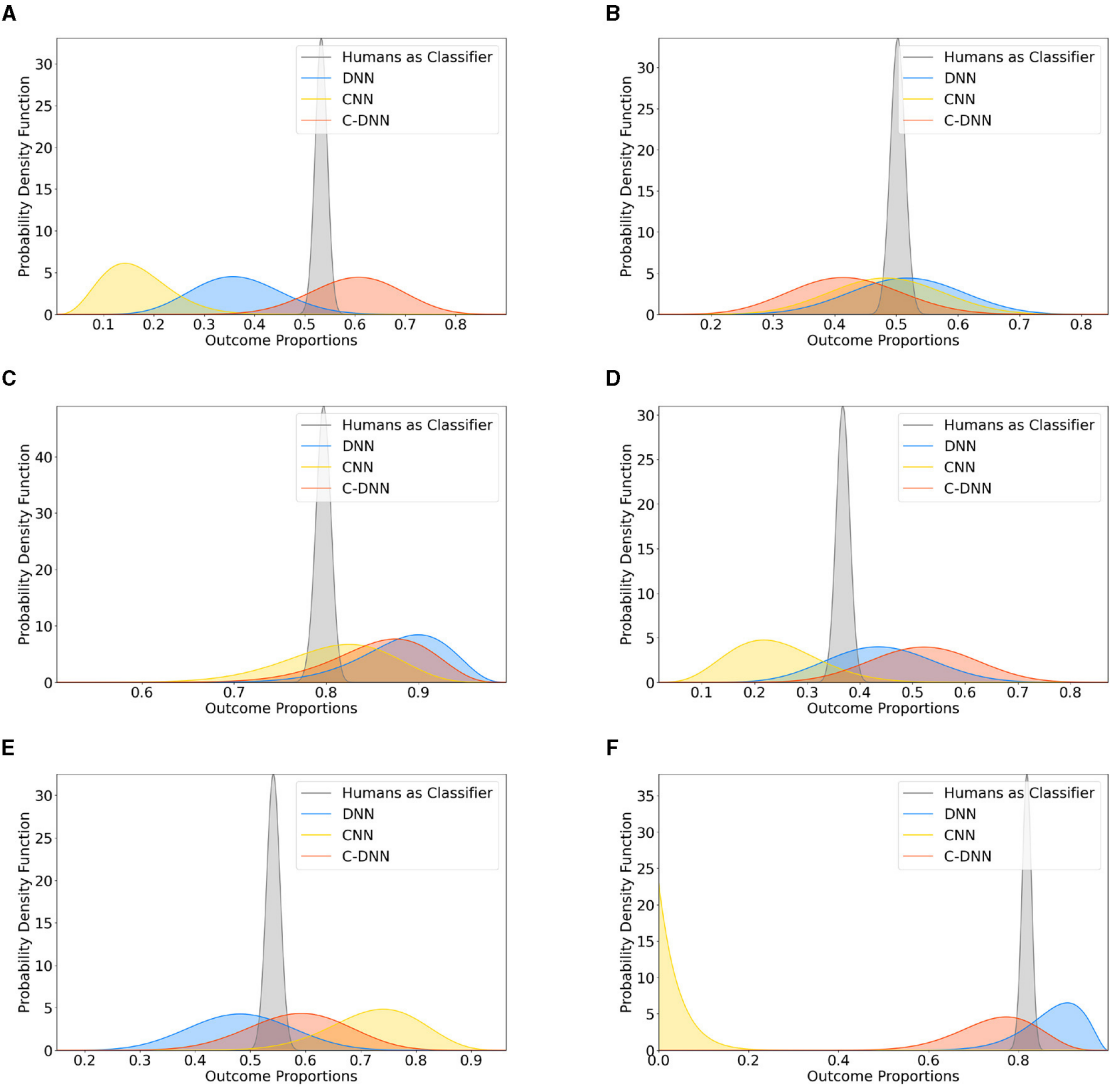
Different neural network designs (DNN, CNN, and C-DNN) compared to human classification across emotions. This figure presents the updated beta distributions for the comparison between different neural network designs (DNN, CNN, and C-DNN) and humans in classifying different emotions. Subfigures correspond to: **(A)** fear, **(B)** joy, **(C)** anger, **(D)** disgust, **(E)** sadness, and **(F)** neutral.

Figure 4 presents a comparative analysis between the three model designs and human performance in classifying the basic emotions and neutral. Each sub-figure corresponds to an emotion, namely, fear A, joy B, anger C, disgust D, sadness E, and neutral F. Both the DNN and the C-DNN design show comparable performance with the participants while the CNN shows unreliable performance across emotions. The sub-figures illustrate the beta distributions of the classifiers' performance. The spread and central tendencies of these distributions provide an understanding of the variance in the performance of the models and the humans.

## Discussion

This article compared the effectiveness of three model designs: Deep Neural Network (DNN), Convolutional Neural Network (CNN), and a combination of the two (C-DNN). Each model was trained and evaluated using three different versions of datasets. The methods of evaluation included 10-fold cross-validation, a combination of Independent Validation and Bayesian Updating, and a comparison with human performance.

The cross-validation revealed the combined model (C-DNN) to be most effective on the combined dataset, while the CNN showed less performance and reliability across all datasets. When a combination of Independent Validation and Bayesian Updating was used, each model performed notably better than random guesses. Nonetheless, the CNN model showed lower performance than its counterparts under all circumstances.

A comparison with human emotional state classification revealed that the DNN and C-DNN models performed at a level similar to humans, whereas the CNN model was less consistent across all emotions.

### Design-specific aspects

The CNN model design in this article showed strong overfitting, leading to poorer and less stable performance than anticipated. This overfitting could be attributed to the segmentation of audios into 1.5 s units, which may have disrupted the emotional structure and limited the models ability to capture nuanced emotional patterns. Future research should explore improving approaches that capture the temporal dynamics of emotions more effectively. For instance, using overlapping windows might be beneficial. This approach would involve half-second increments of audio, providing significant overlap to average out effects. This could potentially capture varying emotional patterns more effectively, even beyond the usual 1.5 s segments.

### Different datasets

When comparing the different datasets, it is evident that all models can predict emotions based on the generated features from audios better than guessing and in the case of the DNN and C-DNN comparatively well as humans. However, the performances vary. The Emo DB dataset consistently leads to the best performance for the DNN design and also for the C-DNN design by excluding the one outlier. It is important to note that this dataset is smaller and less diverse compared to the RAVDESS dataset. Therefore, better generalization of the models cannot be derived from higher performance.

#### RAVDESS and Emo DB combined

The combined dataset from Emo DB and RAVDESS produced comparable performances to the results based on the RAVDESS dataset. Only the CNN design showed inconsistent results, while the other two models showed consistant results.

Although English and German share a common Germanic origin, a uniform language-specific emotional expression cannot be assumed. The consistent performance for the DNN and CNN designs is, therefore, even more remarkable. Despite the limitations of the clipped audio recordings and the heterogeneous datasets, they show a consistent performance across the different datasets used. In particular, considering the comparable performance to the participants, it could be argued that the models have recognized the underlying patterns of emotion contained in the audio recordings beneath the culturally specific facets.

#### RAVDESS and Emo DB separated

It is worth noting that the RAVDESS dataset is significantly larger than the Emo DB dataset and consists of English recordings with a neutral North American accent, which includes Canadian English. Canadian English is characterized by “Canadian Rising,” a phenomenon that affects vowel formants and could impact the acoustic analysis and emotion recognition accuracy. It describes the habit of pronouncing vowels that are normally pronounced with low tongue position with a middle tongue position (Chambers, 1973). The key point is not the change in the word's pronunciation (where vowels sound higher) but the accompanying shift of the vowel formants. This linguistic phenomenon is visible in the acoustic analysis and could thus cause slight irritations with regard to emotion recognition, which are reflected in the performance of the classifier. This aspect could also be a limiting factor for the models based on the combined dataset, as it could prevent further generalization.

### Different models

An integral part of this article was an investigation into whether the combined C-DNN model, leveraging both spectrograms and numerical features, could offer additional informational benefits over the DNN and CNN models used independently. The C-DNN model did exhibit a minor improvement in performance; however, this incremental gain did not proportionally reflect the potential combination of the DNN and CNN models, as one might intuitively expect. This suggests that the added complexity of the C-DNN may not necessarily translate into substantial gains in emotion recognition performance. One possible explanation is that the information in the spectrograms might already be represented in the generated features. Consequently, the additional data from the spectrograms might not enhance the generalization of emotion recognition. Also, the cropping of the audios could have reduced the information value of the spectograms to such an extent that they can no longer reliably represent emotion-related information. Both of these aspects could contribute to the CNN design over-adapting to non-emotion-related aspects or learning culture-specific facets lacking compensation from the features unlike the C-DNN.

### Comparison with previous studies

A classification based on short 1.5 s audio sequences has not been approached in the literature to the authors best knowledge. Short clips of this length are a solution approach when it comes to classifying the emotions to be heard within a longer audio stream without performing complex preprocessing. As can be seen in Table 1, performance for longer audio sequences (in the literature listed there, ranging from 1.5 to 5 s) can allow for higher accuracies. We have deliberately worked with audio files as brief as 1.5 s to highlight the feasibility and potential of real-time emotion recognition in dynamic settings. Longer audio clips might yield more accurate results; however, they are less reflective of actual conditions where audio data is rarely perfect and manually segmenting emotional content is often unfeasible. Our choice of a 1.5 s timeframe aims to emulate an automated system that may imperfectly trim audio segments, thereby mirroring the practical challenges faced by classifiers in real-world applications. These segments are short and concise enough for human comprehension and also represent the minimal length necessary to retain substantial information from the raw audio without introducing uninformative content into the analysis. In addition, models were created for the DNN designs based on differently segmented audio files (3 and 5). As expected, there is a higher accuracy for the 3 s audio files, but no clear increase for the 5 s length. This could be due to the type of audio processing, as the audio files that were too short were lengthened by adding silence. This could, on the one hand, make the classification more difficult and, on the other hand, could require a higher complexity of the models in order to learn the correct patterns. This additional complexity could potentially require more computing power than was employed. It should be highlighted that in this article good performances were achieved on the combined dataset, which was not attempted or reported previously.

The current analyzes show that even on very short audio sequences, classification is well above guessing, comparable to human precision and ranging in the order of magnitude of 50–60% accuracy, which is still low when relying on it for a single subsequence. However, future work based on the tool could use models designed to combine information over time (as for example pooling over time or hidden Markov Chains) to boost the performance. The SHAP values in Figure 3 offer an empirical basis for evaluating the optimal length of audio segments for emotion recognition models. Higher SHAP values in specific segments suggest that these intervals contain critical emotional information. The consistent presence of such segments across datasets could implie that shorter, information-rich audio clips could be sufficient and potentially more effective for training emotion recognition models. Conversely, segments with lower SHAP values may contribute less to model performance, indicating that longer audio recordings could introduce redundancy or noise. These observations highlight the potential for more efficient model training with carefully selected, shorter audio segments that maximize emotional content. Also, in a time series of emotion classification, some errors may not be as problematic as a miss-classification of a longer, complete audio stream would be. Therefore, it seems plausible that the current approach may allow to generate an emotion time series from an audio stream with sufficient precision.

### Comparison with humans

The emotion recognition ability of the models used in this article demonstrated performances comparable to humans, blurring the line between human judgments and model predictions. This suggests that the employed models successfully emulated the human capacity for audio-based emotion recognition in terms of performance. Furthermore, the comparable accuracy between humans and the models implies the involvement of similar mechanisms of pattern recognition.

However, further investigations are required to delve into the intricate workings of the neural network and its alignment with human cognitive processes. This article offers a novel approach to investigate the complexities of audio based human emotion understanding through the application of neural networks. By reverse-engineering such models, valuable insights into the underlying mechanisms and cognitive processes involved in human emotion recognition may be gained. This interdisciplinary research, bridging psychology and computer science, highlights the potential for advancements in automatic emotion recognition and the broad range of applications.

### Limitation

The use of actor-performed emotions as the gold standard for developing classification systems may not capture the full range and authenticity of emotions. Actor-performed emotions may not represent the subtler and more authentic emotions often encountered in everyday situations. Given the current state of the models presented, the use of real-life data is questionable due to the databases used. Developing a new dataset that includes a broader range of emotions and different levels of intensity is, therefore, crucial but poses challenges. Heterogeneous datasets containing emotions of varying intensity from different individuals and diverse acoustic qualities may present difficulties in reliably labeling and classifying emotions.

However, this remains the objective, as classifications would ideally be performed on data that closely mirrors reality. In future research enriching the dataset with a broader spectrum of emotions and cultural backgrounds could improve the models' capabilities to recognize a variety of emotional expressions. The exploration of the role played by linguistic differences in emotion recognition could further improve the performance of the models and enhance their practical application.

The influence of linguistic differences on emotion-specific acoustic patterns are another important aspect to consider. Care must be taken to differentiate between patterns that correlate directly with emotions and those influenced by other factors unrelated to emotions. Specializing the classification system in emotion-specific patterns while being resistant to other voice-related information is crucial. Future investigations could delve into the impact of linguistic variations, such as languages and dialects, on the formation of acoustic patterns. By integrating speech recognition into the classification tool, it may be possible to categorize recordings based on language families or cultural linkages. Given the ability to adequately filter acoustic disruptions, such as ambient noise or white noise, the emotion classifier could extend its applications into diverse realms, ranging from everyday interactions to clinical or therapeutic settings.

In these settings, an amalgamation of tools for classifying vocal and facial emotional expressions might offer added benefits. By simultaneously analyzing voice and facial cues, it could pave the way for the creation of adaptive algorithms that generate tailored classification tools, serving both personal and professional needs regarding a wide variety of emotion-related use cases.

Ekman's theory of basic emotions, while easy to interpret, may oversimplify the complexity of human emotions. Considering multidimensional approaches, such as the one proposed by Fontaine et al. (2007), could provide a more nuanced understanding of emotions by defining them across several dimensions. This would accommodate the intricacies and variability of human emotional experiences, allowing for the representation of intermediate emotional states rather than rigid categories like sadness or joy.

In addition, temporal segmentation of audio material into 1.5 s units could lead to forced emotion recognition because it does not capture the natural flow and temporal dynamics of emotions. For example, the CNN design exhibited overfitting, which could be due to the 1.5 s units used. Investigating alternative methods to better capture the temporal dynamics of emotions could potentially enhance the accuracy and generalizability of these models. One method could involve the usage of overlapping windows of clips instead of separate clips.

In the present study we chose a fixed segment length of 1.5 s. The short segment length allows for a continuous classification of human speech and limits overlapping emotions. And the fixed segment length means that continuous human speech would not need to be preprocessed manually into semantically coherent segments. While these short, fixed segments are, hence, necessary for an automatic continuous classification, it is possible that better accuracies can be achieved with longer time segments, as was found in past studies (e.g., Atmaja and Sasou, n.d.^1^). Future studies should investigate whether the use of longer or shorter segments could be advantageous for, in our case, the recognition ability of humans and classifiers. In regard of optimizing the audio file length in terms of maximizing the accuracy of the models, it could be beneficial to include the length as a continuous variable in the model creation pipeline. It is important to emphasize that the present work does not claim to have used the optimal length with the 1.5 s long segments used. In future research, it is recommended to consider employing on-system interpretable systems like SincNet, along with 1D and 2D convolution approaches (Ravanelli and Bengio, 2018; Mayor-Torres et al., 2021), especially for analyzing multimodal signals, such as the audio in this work, as these methods offer promising avenues for enhanced interpretability and analysis.

Enriching the dataset with a broader spectrum of emotions and cultural backgrounds could improve the models' capabilities to recognize a variety of emotional expressions. The exploration of the role played by linguistic differences in emotion recognition could further augment the models' performance. The application-oriented approach demonstrated in this study opens up possibilities for the development of a standalone software application featuring user-friendly interfaces. This application could make the emotion recognition technology more accessible and relevant for real-world implementation.

### Conclusion

This article presents a novel approach for classifying emotions using audio data. Through the extraction of features from brief 1.5 s audio segments and the employment of diverse models, we achieved accurate emotion classification across all tested datasets. Our Balanced Accuracies consistently surpassed random guessing. Furthermore, the performance metrics of our DNN and C-DNN models closely mirror human-level accuracy in emotion recognition, showcasing their potential. Nevertheless, the CNN models consistently demonstrated inconsistent results across datasets, indicating limited benefits from employing spectrograms.

In future endeavors, it will be imperative to mitigate overfitting, refine the capture of temporal emotional dynamics, and expand the dataset to encompass a wider range of emotions, cultures, and languages. The creation of a standalone software application equipped with user-friendly interfaces could provide an avenue for the wider application of this emotion recognition technology in myriad settings.

## Data availability statement

Publicly available datasets were analyzed in this study. This data can be found at: https://www.kaggle.com/datasets/uwrfkaggler/ravdess-emotional-speech-audio; http://emodb.bilderbar.info/start.html.

## Ethics statement

Ethical review and approval was not required for the study on human participants in accordance with the local legislation and institutional requirements. Written informed consent from the patients/participants was not required to participate in this study in accordance with the national legislation and the institutional requirements.

## Author contributions

HD: Conceptualization, Methodology, Software, Writing – original draft. LS: Conceptualization, Writing – original draft. TB: Writing – review & editing. TO: Methodology, Supervision, Writing – review & editing.

## Footnotes

^1^Atmaja, B. T., and Sasou, A. (n.d.). *Multilingual Emotion Share Recognition From Speech by Using Pre-trained Self-supervised Learning Models*. (unpublished).

## Appendix

**Table A1:** Overview of hyperparameters used for model creation.

**Hyperparameter**	**Options**	**Description**
Number of layer	2 to 8	Describes the depth of the model
Number of neurons	80 to 400	Describes the size of the layers
Activation function	relu	*f*(*x*) = max(0, *x*)
	elu	$f(x)=\{\begin{array}{ccc} \alpha ({\text{e}}^{x}-1) & \text{for} & x\leq 0\\ x & \text{for} & x>0 \end{array}$
	sigmoid	$f\left(x\right)=\frac{1}{1+\exp\left(-x\right)}$
	tanh	$f\left(x\right)=\frac{{\text{e}}^{x}-{\text{e}}^{-}x}{{\text{e}}^{x}+{\text{e}}^{-}x}$
Optimization function	SGD	Optimized using a random training example
	RMSprop	Optimized using a adaptive Learning rate
	Adam	Combination of momentum optimization and adaptive learning rate
Error function	Categorical cross entropy loss	$-\overset{n}{\underset{i}{\sum }}y_{i}\log\left(\hat{y_{i}}\right)$
Learning rate	0.01 to 0.0000001	The step size that the model takes toward minimizing the cost function

This table provides a non-exhaustive overview of the different hyperparameters used in model creation along with their options and descriptions.
